# The role of TNF-α and NFkβ in an experimental model of intestinal carcinogenesis with 1,2-dimethyhydrazine

**DOI:** 10.1590/acb383623

**Published:** 2023-09-18

**Authors:** Márcio Alencar Barreira, Márcio Wilker Soares Campelo, Conceição da Silva Martins Rebouças, Ana Paula Bomfim Soares Campelo, Paulo Roberto Leitão de Vasconcelos

**Affiliations:** 1 Universidade Federal do Ceará – Walter Cantídio University Hospital – Fortaleza (Ceará) – Brazil.; 2 Universidade Federal do Ceará – School of Medicine – Department of Surgery – Fortaleza (Ceará) – Brazil.; 3 Universidade Federal do Ceará – School of Medicine – Department of Morphology – Fortaleza (Ceará) – Brazil.; 4 Centro Universitário Christus – School of Medicine – Fortaleza (Ceará) – Brazil.

**Keywords:** Aberrant Crypt Foci, Carcinogenesis, Colorectal Neoplasms, Tumor Necrosis Factor-alpha, NF-kappa B

## Abstract

**Purpose:**

To analyze the potential of tumor necrosis factor-α (TNF-α) and factor nuclear kappa B (NF-κB) as colorectal cancer (CRC) biomarkers in an experimental model of intestinal carcinogenesis with 1,2-dimethyhydrazine (1,2-DMH).

**Methods:**

Twenty-four male Wistar rats were divided into two groups: sham and 1,2-DMH. First, 1,2-DMH (20 mg/kg/week) was administered for 15 consecutive weeks. In the 25th week, proctocolectomy was conducted. Histopathological analysis, immunohistochemistry, and gene expression of TNF-α and NF-κB were performed. Statistical analysis was performed using GraphPad Prism. The location of aberrant crypt foci (ACF) was analyzed by Kruskal-Wallis’ test. For analyses with two groups with parametric data, the *t*-test was used; for non-parametric data, the Mann-Whitney’s test was used. P < 0.05 was considered significant.

**Results:**

The number of ACF and macroscopic lesions was significantly higher (p < 0.5) in the 1,2-DMH group compared to the sham group, and most ACF were concentrated in the distal segment of the colon. There was a statistically significant increase (p < 0.5) in protein and gene expression of TNF-α and NF-κB in the 1,2-DMH group compared to the sham group.

**Conclusions:**

Our results provide supportive evidence that TNF-α and NF-κB pathways are strongly involved in CRC development in rats and might be used as early biomarkers of CRC pathogenesis in experimental studies.

## Introduction

In general, the incidence and mortality from cancer are growing rapidly worldwide^1^. Malignant neoplasms will be the major cause of morbidity and mortality, surpassing cardiovascular diseases^2^. In men, colorectal cancer (CRC) represents the third neoplasm with the highest incidence (behind lung and prostate cancer) and the fourth neoplasm with the highest mortality (behind lung, liver, and stomach cancer). In women, CRC represents the second neoplasm with the highest incidence (behind breast neoplasm) and the third neoplasm with the highest mortality (behind breast and lung)^3^.

The use of animal models has the potential to increase our understanding of carcinogenesis, tumor biology, and the impact of specific molecular events on colon biology^4^. Rodent models are rapid, reproducible, and exhibit an adenoma-carcinoma sequence like that found in humans^5^. 1,2-dimethyhydrazine (1,2-DMH) is a widely used agent for developing CRC in rodents. Although DMH-induced CRC in rats does not represent the complexity of the human disease and does not replace studies with patient material, it is a valuable tool for studying the molecular events of CRC^6^.

Aberrant crypt foci (ACF) are widely accepted as precursors of CRC morphologically, histologically, biologically, and genetically. Identification of ACF both in carcinogen-treated rodents and in human colon makes the study of CRC at precancerous stages possible. They harbor gene mutations that are vital to tumor formation and progression. By studying these lesions, it may be possible to learn more about the causes of colon carcinogenesis^7^. The prevalence and mean number of ACFs significantly increased with the stage of the adenoma–carcinoma sequence. ACFs may be useful as a reliable surrogate biomarker for colorectal carcinogenesis^8^.

Increased inflammatory stress stimulates adenomatous cell growth^9^ and suggests a strong association between intestinal inflammation and CRC^10^. Research on tumor necrosis factors alpha (TNF-α) and factor nuclear kappa B (NF-κB) have been tightly intertwined. Cytokines belonging to the TNF-α family induce rapid transcription of genes regulating inflammation, cell survival, proliferation, and differentiation, primarily through activation of the NF-κB pathway^11^. NF-κB is increasingly recognized as a crucial player in many steps of cancer initiation and progression^12^.

TNF-α and NF-κB seem to be interesting markers of intestinal carcinogenesis. However, we did not find scientific works dedicated to showing this association. Consolidation of markers related to CRC development strengthens the CRC induction model with 1,2-DMH. Thus, this study aimed to analyze the potential of TNF-α and NF-κB as a CRC biomarker in an experimental model of intestinal carcinogenesis with 1,2-DMH.

## Methods

The sample consisted of 24 male Wistar rats from the Centro Universitário Christus vivarium, weighing 80 ± 10 g. The study protocol was approved by the Ethics and Animal Research Committee (no. 031/2018). The rats were kept in polypropylene cages in a temperature-controlled environment (22 ± 1°C) with a 12-h light/dark cycle, free access to drinking water, and a standard chow diet.

### Experimental design

The rats were randomly divided into two groups of 12 animals each (sham and 1,2-DMH). The rats in the experimental group received a weekly subcutaneous injection of 1,2-DMH (D161802; Ph 6,5; Sigma-Aldrich) at the dose of 20 mg/kg body weight for 15 weeks^13^. The carcinogen was dissolved in 0.9% NaCl, and the control group received the equivalent dose of 0.9% NaCl without adding the carcinogen.

### Surgical procedure and sample preparation

At the end of 25 weeks, the animals were anesthetized with 10% ketamine hydrochloride (80 mg/kg/weight) and 2% xylazine hydrochloride (10 mg/kg/weight). The animals were positioned in dorsal decubitus on a wooden board. Then, a laparotomy and proctocolectomy were performed. Subsequently, the surviving animals were sacrificed by hypovolemic shock after a section of the abdominal aorta.

The proctocolectomy product was opened longitudinally at the antimesenteric border for intestinal lavage and extended with the exposed mucosa. After dividing the colon into three equal segments, the specimens were randomly stored in a 10% buffered formalin solution (n = 12) and a freezer at -80°C (n = 12) without RNA/DNA preservation solution. Samples separated for histology and immunohistochemistry were embedded in paraffin using a conventional method and stained with hematoxylin and eosin (H&E). Then, the paraffin blocks were used to make new slides that were stained with methylene blue (MB) at a concentration of 0,1%^14^. In the frozen samples, the colon’s distal segments were used for polymerase chain reaction (PCR).

### Macroscopy

The presence of macroscopic lesions (ML) was identified and recorded. ML were counted per rat immediately after euthanasia, and the results are shown as an average absolute number per group.

### Microscopy

The colonic mucosa was evaluated with an optical microscope with 20X and 40X objective magnification. To evaluate the slides stained with MB, 10 fields per bowel segment (distal, middle, and proximal) were photographed randomly at 400x magnification. The crypts were analyzed in cross-section, and the analyzed factors were the number of ACF and the location in the colon (distal, middle, and proximal). ACF was considered when the crypts had at least two of the criteria: increased crypt size, thicker epithelial layer, more intense staining (due to nuclear increase and mucin depletion), increased pericrypt zone, and elliptical shape^14^,^15^.

### Immunohistochemistry

Distal colon fragments were collected from each rat from paraffin blocks. Colon and positive control (colon carcinoma) tissue were deposited on silanized glass slides for conventional H&E staining and immunohistochemistry reactions. Immunohistochemistry for TNF-α and NF-κB proteins was performed using the streptavidin-biotin-peroxidase method^16^. In this technique, the slides were deparaffinized, hydrated in xylene and alcohol, and immersed in a retrieval solution of acid or basic pH. Then, antigenic retrieval took place for 30 minutes at 95°C in an automated medium (PT-LINK). After cooling, washings were performed with Dako wash buffer solution, interspersed with blocking endogenous peroxidase with 3% H_2_O_2_ solution (20 min).

Sections were incubated for 1 hour with primary goat Anti-NF-κB p65 (1:200, code ab16502, Abcam, Cambridge, MA, United States of America) and TNF-α (1:100, code ab1793, Abcam, Cambridge, United Kingdom) diluted in antibody diluent. After washing the sections in wash buffer solution, incubation was performed with HRP polymer (Dako) for 30 minutes. The sections were washed again with a wash buffer, followed by staining with the chromogen 3,3’diaminobenzidine-peroxide (DAB) and counterstaining with Mayer’s hematoxylin. Finally, samples were dehydrated, and slides mounted. Negative controls were processed simultaneously and incubated with serum diluent only.

The images were captured using a light microscope coupled to a camera with a LAZ 3.5 acquisition system (Leica DM1000, Germany). Ten fields were photographed per histological section (40x objective), trying to select the areas with the highest marking in each animal (hot areas). For counting positive cells marked by each field, the Adobe Photoshop 8.0 program was used to obtain the total tissue area and immunostained area. Positive cells stained with brown cytoplasmic staining were considered. Then, to measure the percentage (%) of the marked area, Eq. 1 17 was performed:


Marked area (%) = immunomarker area (pixels) × 100 / total area (pixels) (1)

### Quantitative real-time polymerase chain reaction

The gene expression of TNF-α and NF-κB was analyzed in distal colon tissue stored at -80°C. Total RNA was extracted by an RNA isolation system (Promega) according to the manufacturer’s protocol. The RNA was quantified by a NanoDrop spectrometer (Promega Corporation, Madison, WI, United States of America), and RNA quality was determined by examining the 260/280 ratio. A total of 1 μg RNA was then reverse transcribed using a high-capacity cDNA reverse transcription kit (Applied Biosystems; Warrington, United Kingdom) according to the manufacturer’s protocol. qPCR was performed using SYBR Green PCR Master Mix (A25742; Applied Biosystems; Warrington, United Kingdom), as described in the manufacturer’s instructions.

To compare gene expression under different conditions, the expression under each condition (normalized to glyceraldehyde 3-phosphate dehydrogenase—GAPDH, the endogenous control) was quantified relative to the control condition. TNF-α and NF-κB qPCR amplification was performed in a CFX Connect system (Bio-Rad Laboratories, StepOne Real time PCR System, 4376357, United States of America) under the following conditions: 50°C for 2 min and 95°C for 10 min followed by 40 cycles of 95°C for 15 s and 60°C for 60 s. The relative expression levels of the genes were calculated using the threshold cycle (2–ΔΔCT) method^18^. Table 1 shows the sequence of the primers used.

**Table 1:** GAPDH, NF-kβ and TNF-α primer sequences.

Primer	Forwards primer	Reverse primer
GAPDH	5´TGCTGGTGCTGAGTATGTCG3’	5´TTGAGAGCAATGCCAGCC3’
NFK-B	5´CAGAGCTGGCAGAGAGACTG3’	5´TACGAAGGAGACTGCCACTG3’
TNF-α	5´GAGGTCAACCTGCCCAAGTA3’	5´GTCGCTTCACAGAGCAATGA3’

Source: Elaborated by the authors. GAPDH: glyceraldehyde 3-phosphate dehydrogenase; NF-kβ: factor nuclear kappa B; TNF-α: tumor necrosis factor-α.

### Statistical analysis

Statistical analysis was performed using the GraphPad Prism version 6.0 (GraphPad Software Inc., La Jolla, CA, United States of America). The data normality was analyzed using the Shapiro-Wilk’s test. The results of the location of ACFs were analyzed by Kruskal-Wallis’ test, followed by Dunn’s test (multiple comparisons). For analyses with two groups with parametric data, the *t*-test was used; for non-parametric data, the Mann-Whitney’s test was used. P < 0.05 was considered significant.

## Results

**Figure 1:**
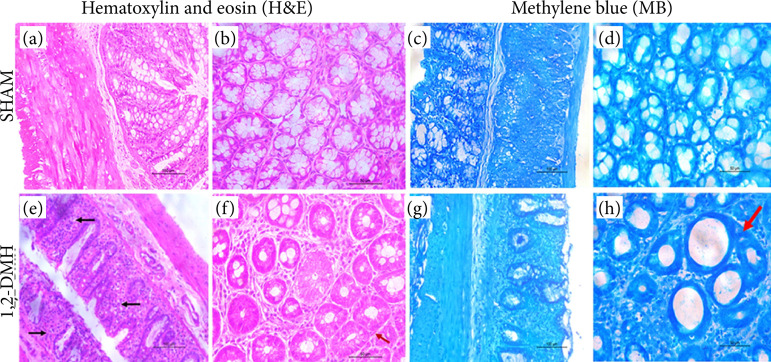
Histopathology of the colon. (**a** and **c**) Longitudinal section with normal crypts showing preserved goblet cells and enterocytes, absence of inflammatory infiltrate, and edema in the mucosa, submucosa, and muscle layer (20x objective magnification; scale bar 100 μm). **(b** and **d**) Cross section with crypts without morphological changes (40x objective magnification; scale bar 50 μm). (**e** and **g**) Longitudinal section with aberrant crypts with inflammatory cell infiltrate (black arrow), loss of goblet cells, shortened and aberrant crypts (20x objective magnification; scale bar 100 μm). (**f** and **h**) Cross-section showing the presence of multiple aberrant crypts (red arrow), increased pericrypt zone, presence of inflammatory cells, reduced goblet cells, and mucin depletion (40x objective magnification; 50 μm scale bar).

Source: Elaborated by the authors.

**Figure 2:**
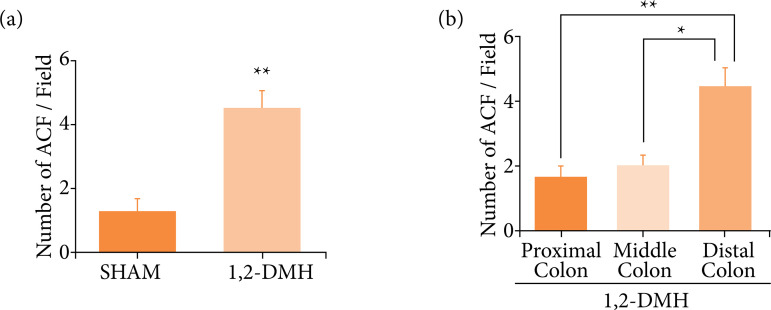
Number of ACFs in the colon and location of ACFs in the 1,2-DMH group. **(a)** Data were analyzed by *t*-test with Mann-Whitney’s test, where **p<0.01 vs. Sham group. **(b)** Data were analyzed by Kruskal-Wallis’ test, followed by Dunn’s test, where *p < 0.05, **p < 0.01 vs. other groups.

Source: Elaborated by the authors. ACF: aberrant crypt foci; 1,2-DMH: 1,2-dimethyhydrazine.

Figure 1 shows the representative images of colon sections stained with H&E and MB from the control group (Figs. 1a–1d) and 1,2-DMH group (Figs. 1e–1h).

In Fig. 2a, the number of ACF was significantly higher (p < 0.01) in the 1,2-DMH group compared to the sham group. Most lesions are in the distal colon, with the difference being more significant (p < 0.01) between the proximal and distal parts of the colon (Fig. 2b).

**Figure 3:**
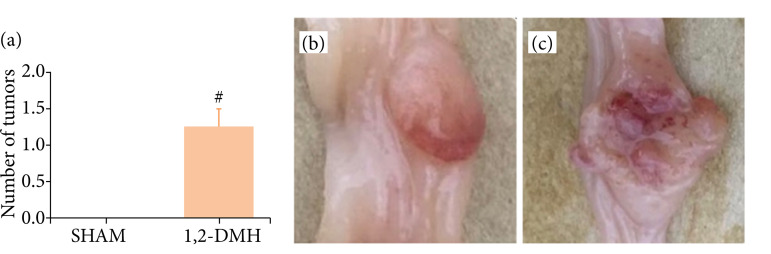
Number and appearance of ML in the colon. **(a)** Graphic representation of the number of ML in the resected specimen. Data were analyzed by *t*-test with Mann-Whitney’s test, where #p < 0.05 vs. sham. (b) ML with a polypoid appearance. (c) ML with an ulcerated appearance.

The 1,2-DMH group showed a significant increase in the number of ML compared to the sham group (p<0.05) (Fig. 3a). Figures 3b and 3c show examples of ML with distinct characteristics accounted for in the macroscopic evaluation.

**Figure 4:**
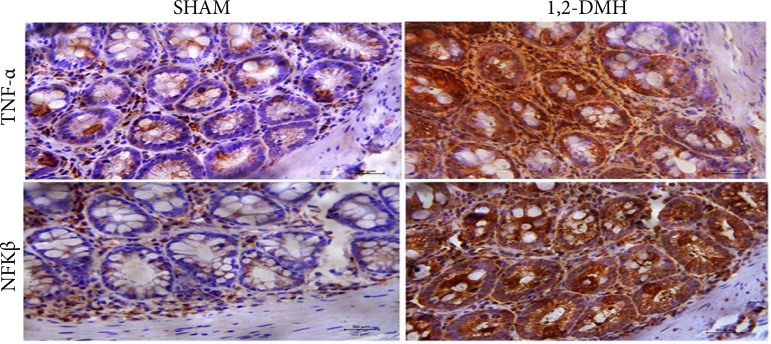
Expression of TNF-α and NF-Kβ proteins in the distal colon of Wistar rats. The photos are with 40× objective magnification (50 μm scale bar), cross section, and the staining used in the tissue was MB. Data were analyzed by *t*-test with Mann-Whitney’s test.

Source: Elaborated by the authors. TNF-α: tumor necrosis factor-α; NF-Kβ: factor nuclear kappa B; MB: methylene blue; *p < 0.05, **p < 0.01 vs. sham group.

There was a statistically significant increase (p < 0.5) in protein expression of TNF-α and NF-κB in the 1,2-DMH group compared to the sham group (Fig. 4).

**Figure 5:**
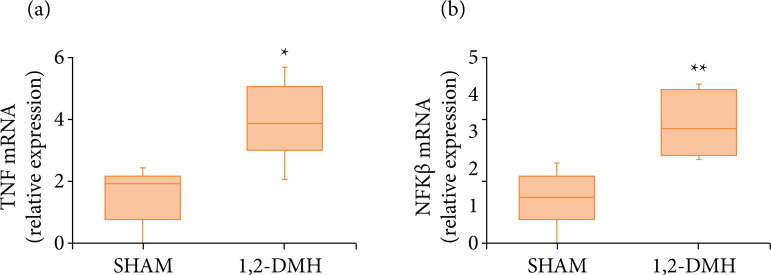
Expression of TNF-α and NF-Kβ genes in the distal colon of Wistar rats. Data were analyzed by *t*-test with Mann-Whitney’s test.

Source: Elaborated by the authors. TNF-α: tumor necrosis factor-α; NF-Kβ: factor nuclear kappa B; *p < 0.05, **p < 0.01 vs. sham group.

There was a statistically significant increase (p < 0.5) in gene expression of TNF-α and NF-κB in the 1,2-DMH group compared to the sham group (Fig. 5).

## Discussion

The Wistar rat can be used in the study of colorectal carcinogenesis, but it requires more prolonged exposure to the carcinogen^19^. Transgenic mice or rats are also widely used to study colon tumorigenesis. Transgenic mice with adenomatous polyposis coli (APC) mutation develop CRC more quickly. However, tumors developed in these models are mostly located in the small intestine instead of the colon^20^. Nowadays, the DMH model represents a useful research tool for the study of colonic carcinogenesis. The carcinogenic effect may be obtained after a single injection or via a series of weekly injections. The tumor incidence can be modulated by the amount of carcinogen administered and the number of applications^21^.

Bird was the first to identify and describe ACF in the colorectal mucosa of rodents exposed to the action of carcinogenic substances, in addition to recognizing them as an early lesion and precursor of CRC^14^. Subsequently, ACF was recognized and identified in humans^22^ and has since shown its potential as a reliable biological biomarker of preneoplastic and cancerous lesions in the large intestine^23^. Sakai et al.^8^ performed high magnification chromoscopic colonoscopy to identify ACF in 861 subjects undergoing a diagnostic endoscopy. The study compared the number of ACF in three subject groups (normal subjects, adenoma cases, and CRC cases). The mean number of ACF was 3.6, 6.2, and 10.1, in normal subjects, adenoma cases, and CRC cases, respectively. As expected, our study confirmed a higher number of ACFs and ML in the 1,2-DMH-induced CRC group compared to the sham rats.

Braga et al.^24^ experimentally induced intestinal carcinogenesis. and after 16 weeks the ACF were quantified. The number of ACF was higher in the distal colon (43.17 ± 16.46) compared to the proximal colon (2.33 ± 3.83) and middle colon (23.83 ± 18.00). The incidence of ACF is higher in the distal segment of the colon^8^, ^25^. Our data indicated that the highest number of ACF was in the distal segment of the colon.

TNF-α levels are one of the most used parameters to characterize experimental models of colitis-associated CRC^26^. The combination of 1,2-DMH (30 mg/kg) with dextran sulfate sodium (seven days) has been used for the induction of CRC in rats, and an increase in TNF-α levels was highlighted in the cancer group^27^. Elevated serum levels of TNF-α show a marked relationship with the increased risk of colorectal adenomas in men^28^, and suppressing TNF-α signaling reduced the number and size of polyps in an animal model^29^.

The inappropriate or excessive activation of TNF-α signaling is associated with chronic inflammation and can eventually lead to the development of a wide variety of diseases^30^. However, our data showed that during CRC induction in rats with 1,2-DMH the high TNF-α tissue levels were markedly associated with CRC precursor lesions.

A myriad of genes regulated by NF-κB transcription factors has been shown to mediate inflammation, cellular transformation, tumor cell survival, proliferation, angiogenesis, and metastasis^31^. The reason why NF-κB is constitutively and persistently active in cancer cells is not fully understood, but multiple mechanisms have been described. Activation of this signaling pathway can occur through a specific agent (viruses, proteins, bacteria, and cytokines), signaling intermediates (mutant receptors, kinase overexpression, mutant oncoproteins, histone deacetylase, and induced nitric oxide sinthase), and crosstalk between NF-κB and other transcription factors (STAT3, β-catenin, p53)^32^. In mice models, the NF-κB pathway has been directly linked to intestinal inflammation and to the development of colitis-associated cancer^33^. The expression of NF-κB in the colonic tissue of patients with CRC, inflammatory bowel disease, and polyps was evaluated, and a higher expression of NF-κB was observed in patients with CRC. This finding may support the hypothesis that NF-κB plays an important role early in the process of colonic dysplasia development that may lead to cancer^34^.

One of the limitations of this study is that we could not dissect the histopathological changes seen in the ML in more depth. Thus, polypoid and tumor-like lesions were counted in the same way. Some studies have revealed significant associations between the number of ACF and the synchronous presence of advanced neoplasms, including both adenoma and CRC^35^,^36^.

The TNF-α and NF-κB influence on Wnt/β-catenin signaling pathway may be further explored in future studies since this pathway is considered one of the first events in the development of CRC^37^. In addition, other TNF-α and NF-κB-related signaling pathways controlling cell cycle and tumorigenesis may shed light better to understand the early pathophysiology of intestinal carcinogenesis induction.

## Conclusion

Our results provide evidence that the TNF-α and NF-κB pathways are strongly involved in CRC development in rats and might be used as early biomarkers of CRC pathogenesis in experimental studies.

## Data Availability

All datasets were generated or analyzed in the current study.
